# Supplementary material for: Antimicrobial Resistance in Bacterial Strains of Agricultural Interest: Predictions Based on Genomic Data
Source: Antibiotics (Basel). 2025 Dec 20;15(1):14. doi: 10.3390/antibiotics15010014 (PMC12838024; doi:10.3390/antibiotics15010014)
Supplement: Supplementary file 1 [file antibiotics-15-00014-s001.zip › antibiotics-4006076-supplementary.pdf]

SUPPLEMENTARY INFORMATION

Supplementary Information Figure S1. Antimicrobial susceptibility test with disks. Example of squared plates showing the realization of the antimicrobial susceptibility test based on disks. Nine antibiotics were tested. In this case, the strain MHA1 (Gram positive) is shown.

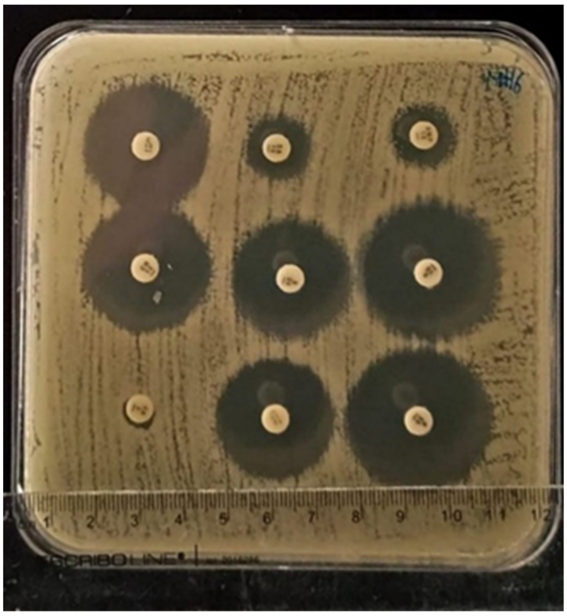

Supplementary Information Figure S2. Determination of MIC using the broth microdilution test. For strain MHA1 the MIC for this antibiotic was 16 mg/L whereas for N23 the MIC was 64 mg/L.

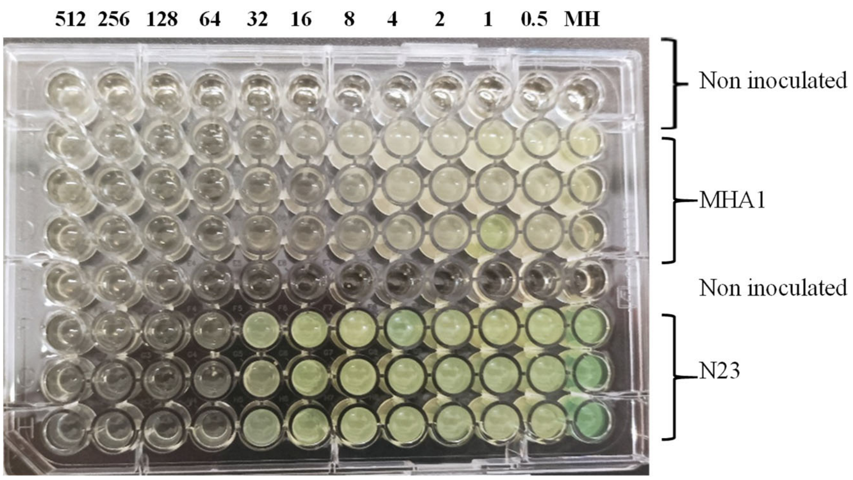

Supplementary Information Figure S3. Determination of the MIC for Fosfomycin using the agar dilution test in Müeller-Hinton agar supplemented with 25 mM glucose-6-phosphate. For the strain N25, the red arrow shows the presence of growth and the blue arrow shows the absence of growth, indicating that for this strain the MIC for fosfomycin was 256mg/L.

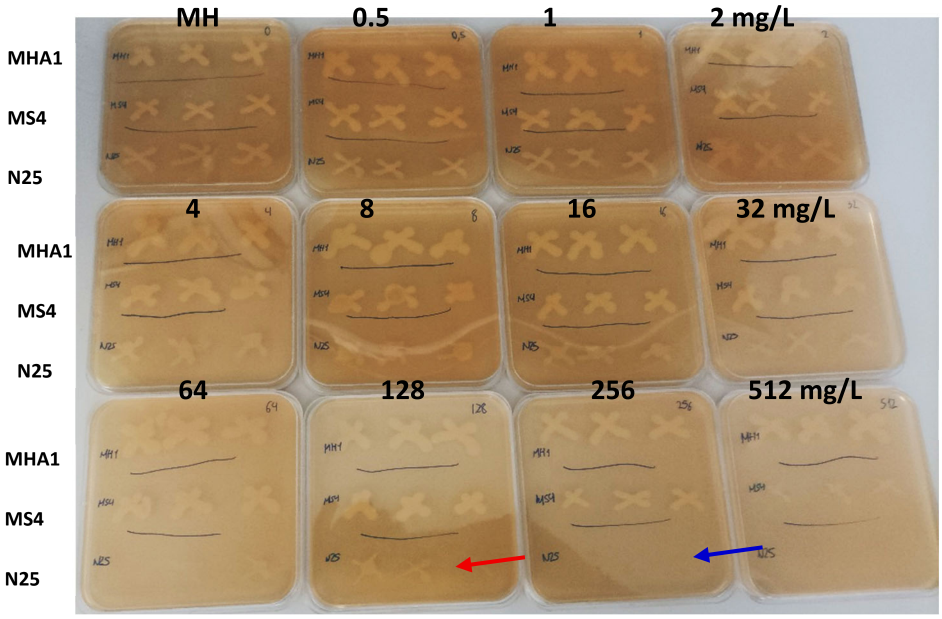
MH
0.5
1
2 mg/L
4
8
16
32 mg/L
64
128
256
512 mg/L
MHA1
MS4
N25
MHA1
MS4
N25
MHA1
MS4
N25
